# Death and End-of-Life Care in Emergency Departments in the US

**DOI:** 10.1001/jamanetworkopen.2022.40399

**Published:** 2022-11-04

**Authors:** Jonathan Elmer, Nancy Mikati, Robert M. Arnold, David J. Wallace, Clifton W. Callaway

**Affiliations:** 1 Department of Emergency Medicine, University of Pittsburgh, Pittsburgh, Pennsylvania; 2 Department of Critical Care Medicine, University of Pittsburgh, Pittsburgh, Pennsylvania; 3 Department of Neurology, University of Pittsburgh, Pittsburgh, Pennsylvania; 4 Department of Medicine, Division of Palliative Care and Medical Ethics University of Pittsburgh, Pittsburgh, Pennsylvania

## Abstract

**Importance:**

There are more than 140 million annual visits to emergency departments (EDs) in the US. The role of EDs in providing care at or near the end of life is not well characterized.

**Objective:**

To determine the frequency of death in the ED or within 1 month of an ED visit in an all-age, all-payer national database.

**Design, Setting, and Participants:**

The retrospective cohort study used patient-level data from the nationally representative Optum clinical electronic health record data set for 2010 to 2020. Data were analyzed from January to March 2022.

**Exposures:**

Age, Charlson Comorbidity Index (CCI), and year of ED encounter.

**Main Outcomes and Measures:**

The primary outcome was death in the ED, overall and stratified by age, CCI, or year. A key secondary outcome was death within 1 month of an ED encounter. We extrapolated to make national estimates using US Census and Nationwide Emergency Department Sample data.

**Results:**

Among a total of 104 113 518 individual patients with 96 239 939 ED encounters, 205 372 ED deaths were identified in Optum, for whom median (IQR) age was 72 (53 to >80) years, 114 582 (55.8%) were male, and 152 672 (74.3%) were White. ED death affected 0.20% of overall patients and accounted for 0.21% of ED encounters. An additional 603 273 patients died within 1 month of an ED encounter. Extrapolated nationally, ED deaths accounted for 11.3% of total deaths from 2010 to 2019, and 33.2% of all decedents nationally visited the ED within 1 month of their death. The proportion of total national deaths occurring in the ED decreased by 0.27% annually (*P* for trend = .003) but the proportion who died within 1 month of an ED visit increased by 1.2% annually (*P* for trend < .001). Compared with all ED encounters, patients with visits resulting in death were older, more likely to be White, male, and not Hispanic, and had higher CCI. Among ED encounters for patients aged older than 80 years, nearly 1 in 12 died within 1 month.

**Conclusions and Relevance:**

This retrospective cohort study found deaths during or shortly after ED care were common, especially among patients who are older and with chronic comorbidities. EDs must identify patients for whom end-of-life care is necessary or preferred and be equipped to deliver this care excellently.

## Key Points

**Question:**

What is the frequency with which US residents die in the emergency department (ED) or within 1 month of an ED visit?

**Findings:**

In this cohort study of a national all-payer database covering 104 113 518 individual patients with 96 239 939 ED encounters, ED deaths accounted for 11.3% of total deaths in the US and 33.2% of all decedents visited the ED within 1 month of their death. These deaths were more common during or after ED visits by patients who were older and those with more comorbidities.

**Meaning:**

These findings suggest emergency medicine practitioners must be able to identify patients for whom end-of-life care is necessary or preferred and have the resources necessary to delivery this care.

## Teaser

This cross-sectional study evaluates the frequency with which US residents die in the emergency department or within 1 month of vising the emergency department.

## Introduction

There are more than 140 million visits to emergency departments (EDs) in the US each year. EDs are intended and designed to provide prompt care for unexpected and unscheduled medical situations. Since emergencies sometimes result in death, all EDs must prepare for this eventuality. Although the number of deaths in EDs decreased from 1997 to 2011,^1^ other data suggest that ED visits for care at or near the end of life are increasing.^2^ From 1992 to 2006, it is estimated one-half of patients older than 65 years visited an ED in the last month of life.^3^

It is not clear what additional resources are needed for end-of-life care in the ED or whether EDs are a good location for patients when death may be anticipated. For example, patients with cancer who die in the intensive care unit have a lower quality experience, including more physical and psychological distress, compared with those who die at home.^4^ Furthermore, the families of patients who die in intensive care have a higher risk of prolonged grief disorders.^5^ Similar results may occur during ED encounters that involve imminent or actual death. Systems for health care delivery that increase outpatient resources or promote planning might help prevent some patients from coming to the ED near the end-of-life. For those patients who do present to the ED at the end of life, designing better support or access to alternative locations of care might reduce patient or family distress.

We aimed to better characterize the epidemiology of end-of-life care in US EDs, and thus quantify the opportunity for deploying interventions or system supports, using a large electronic health record (EHR) database. Our specific objective was to quantify the incidence of death in the ED or within 1 month of an ED encounter. We compared these cases with ED encounters overall to identify patient factors associated with ED utilization at the end of life. Finally, we extrapolated our findings to estimate the frequency of death in the ED or within 1 month of an ED encounter in the US.

## Methods

### Study Design and Data Sources

The University of Pittsburgh Human Research Protection Office approved this study. No written informed consent was required for analysis of deidentified data sources, in accordance with 45 CFR §46. We followed the Strengthening the Reporting of Observational Studies in Epidemiology (STROBE) reporting guideline for cohort studies.^6^ We performed a retrospective cohort study using the Optum clinical EHR data set (Optum EHR) for 2010 to 2020. Optum EHR is a fully deidentified nationally representative data set that contains granular patient-level data extracted from inpatient and outpatient EHRs of patients treated at contributing institutions. Nearly one-third of US hospitals currently contribute data to Optum EHR. Unlike claims-based data, Optum EHR includes patients regardless of age or insurance status. Individual patients are assigned a unique identifier allowing health status and utilization to be tracked over time. Data available include patient demographics, vital status, and month of death extracted from the National Death Index, details of inpatient and outpatient health care encounters, and diagnoses, among other data elements.

We obtained national data for comparison from separate databases. We used data from the Healthcare Cost and Utilization Project Nationwide Emergency Department Sample (NEDS) to determine annual total number of ED visits by US Census region from 2010 to 2020. We used US Census data to determine total number of deaths in the US annually. We used all available data and did not perform an a priori sample size calculation.

### Overall ED Patients

We identified the total number of unique patients in Optum EHR, the number of patients with at least 1 ED encounter, and the total number of ED encounters. New encounters are automatically generated by contributing hospitals’ EHRs, such that multiple apparent encounters may be generated for a single patient over the course of minutes or hours due to human variation in ED registration, ED-to-ED transfer, or other anomalies. To account for this, we considered multiple ED encounters within a 24-hour period to reflect a single care episode.

### Cohorts for Analysis

We used a stepwise approach to identify the cohort of patients who died in the ED. To ensure full deidentification, Optum EHR records vital status by calendar month rather than by day. Since Optum does not include “deceased” as a discharge disposition category for ED, we identified ED encounters with a disposition of “Other” or “Not recorded” as proxy categories. Of these patients, more than 99% died during the same calendar month, which validates our choice of proxies. From this group, we excluded any remaining patients with clinical encounters more than 2 calendar days after the ED encounter under consideration. We also excluded patients who were transferred out of the ED to another care area in the hospital, including those who died after physically departing the ED. By contrast, we included patients who were admitted to the hospital from the ED but died before physical departure to another clinical area within the hospital (ie, admitted patients who were awaiting transfer out of the ED or considered to be boarding in the ED).

We developed 2 comparison groups from Optum EHR. First, we considered patients who survived to disposition from their last ED encounter but died within 1 calendar month of that encounter. Second, we considered all ED encounters. For computational feasibility, to determine chronic comorbidities we selected a random sample of 5% of ED visits. From this sample, we also identified encounters resulting in death in the ED or death within 1 calendar month.

### Data Elements

We extracted patient demographics including sex, race (African American, Asian, White, or Other [American Indian or Alaska Native; Native Hawaiian or Other Pacific Islander]), ethnicity (Hispanic, not Hispanic, or unknown), and US Census region of residence (as categorized by the US Census Bureau^7^). Race and ethnicity were extracted from source hospitals' electronic health records and assessed because of potential differing patterns of ED utilization across these social determinants of health. Optum includes patient birth year for patients born after 1931 (ie, aged >80 years). We calculated age as the interval in years from birth year to encounter, then categorized age into infants (<1 year), pediatrics (1 to 17 years), and then aggregated by decade. For each patient, we identified all diagnoses dated before the ED encounter under consideration. We used the icd package in R statistical software version 4.0.9 (R Project for Statistical Computing) to map diagnoses to categories with the Charlson Comorbidity Index (CCI), and to calculate each patient’s total CCI.^8,9^

### Statistical Analysis

First, we described the patient characteristics across each cohort. We used descriptive statistics to summarize demographic characteristics of each patient cohort and report raw numbers with corresponding percentages or medians with IQR. We used tests of proportions to calculate differences in categorical variables with associated 95% CIs and Hodges-Lehmann median difference test to calculate differences in medians with associated 95% CIs.

Second, we extrapolated from the Optum EHR data to the national totals for ED encounters from NEDS to estimate the total number of ED deaths and deaths within 1 month of an ED visit for the US. Our extrapolation was weighted by US Census region and year, to account for time-varying regional rates of sampling within Optum EHR. We assumed across region and year that mortality in the ED or within 1 month was not different between hospitals participating in Optum and hospitals not participating in Optum. We used simple linear regression to test for trends in the proportion of deaths occurring in the ED or within 1 month of last ED visit over time. In the sample of overall ED encounters, we calculated the proportion with death in the ED or within 1 month, stratifying by age and CCI. As a post hoc analysis, we examined trends in ED death among encounters by patients with a medical history of congestive heart failure or chronic obstructive pulmonary disease.

We performed all analyses using R version 4.0.9 (R Project for Statistical Computing) and considered results to be significant at a 2-sided level of *P* < .05. Data were analyzed from January to March 2022.

## Results

We identified 205 372 ED deaths in Optum, for whom median (IQR) age was 72 (53 to >80) years, 114 582 (55.8%) were male, and 152 672 (74.3%) were White. These patients were identified from a total of 104 113 518 individual patients in the Optum EHR data set, of whom 32 358 675 (31%) had at least 1 ED encounter from 2010 to 2020, and 96 239 939 unique ED encounters in Optum, corresponding to 6.4% of the total estimated ED visits in the US. ED death occurred in 205 372 of 104 113 518 (0.20%; 95% CI, 0.20%-0.20%) patients and accounting for 205 372 of 96 239 939 (0.21%; 95% CI 0.21%-0.21%) ED encounters. We identified an additional 603 273 patients who left the ED alive but died within 1 month of an ED encounter. We found that more than 1 in 3 US residents come to the ED within 1 month of their death, a proportion that has increased 42% over a decade, from 665 254 individuals (26.9%) in 2010 to 1 089 559 individuals (38.2%) in 2019 (Table 1).

**Table 1.** Estimated Annual Total Number and Proportion of Deaths Occurring in the ED or Within 1 Month of ED Encounter in the US

Year	Deaths, No. (%)		Total deaths, No.^b^
	Occurring in the ED	Occurring ≤1 mo from ED encounter^a^	
2010	324 364 (13.1)	665 254 (26.9)	2 468 535
2011	315 294 (12.5)	699 068 (27.8)	2 515 458
2012	290 494 (11.4)	754 650 (29.7)	2 543 279
2013	297 621 (11.5)	804 110 (31.0)	2 596 993
2014	291 292 (11.1)	903 907 (34.4)	2 626 418
2015	309 791 (11.4)	967 324 (35.7)	2 712 630
2016	296 366 (10.8)	998 120 (36.4)	2 744 248
2017	294 586 (10.5)	1 022 089 (36.3)	2 813 503
2018	309 655 (10.9)	983 117 (34.6)	2 839 205
2019	289 453 (10.1)	1 089 559 (38.2)	2 854 838

Abbreviation: ED, emergency department.

^a^ Data were obtained from Optum electronic health record data and total ED visits from Nationwide Emergency Department Sample.

^b^ Data were obtained from US Census data.

There were multiple demographic differences between patients who died in the ED and the population of ED patients overall, and between patients who died within 1 month of their final ED visit and ED patients overall (Table 2). Compared with all ED patients, those with ED death or death within 1 month of an ED visit were older (median [IQR] age, 36 [22 to 57] years vs 72 [53 to >80] years and 73 [54 to >80] years, respectively) and were more likely to be male (42 259 792 [43.9%]; 95% CI, 43.9%-43.9%) vs 114 582 (55.8%; 95% CI, 55.7%-56.0%) and 312 351 (51.8%; 95% CI, 51.6%-51.9%), respectively. Among ED encounters for patients older than 80 years, nearly 1 in 12 died within 1 month of that encounter. Each individual chronic comorbidity was more common among both categories of decedents than the general ED population (Table 3). Among ED encounters overall, the frequency of ED death and death within 1 month varied across age (0% to 1.9% for ED death; 0% to 8.1% death within 1 month) and CCI (0% to 1.3% ED death; 0.2% to 4.3% death within 1 month) (Figure).

**Table 2.** Characteristics of Patients With at Least 1 ED Visit, ED Death, or Death Within 1 Month From Last ED Encounter

Characteristic	Participants, No. (%)				
	ED visits overall (n = 96 239 939)	ED deaths (n = 205 372)	Difference in proportions vs ED patients overall, mean % (95% CI)	Death ≤1 mo from ED encounter (n = 603 273)	Difference in proportions vs ED patients overall, mean % (95% CI)
Age, y					
<1	1 060 335 (1.1)	1342 (0.7)	−0.4 (−0.4 to −0.4)	1071 (0.2)	−0.9 (−0.9 to −0.9)
1-17	17 368 185 (18.0)	2789 (1.4)	−16.7 (−16.7 to −16.6)	4808 (0.8)	−17.2 (−17.3 to −17.2)
18-29	20 402 061 (21.2)	6764 (3.3)	−17.9 (−18.0 to −17.8)	12 015 (2.0)	−19.2 (−19.2 to −19.2)
30-39	14 959 535 (15.5)	8310 (4.1)	−11.5 (−11.6 to −11.4)	15 856 (2.6)	−12.9 (−13.0 to −12.9)
40-49	12 208 469 (12.7)	12 438 (6.1)	−6.6 (−6.7 to −6.5)	27 187 (4.5)	−8.2 (−8.2 to −8.1)
50-59	11 541 929 (12.0)	25 029 (12.2)	0.2 (0.1 to 0.3)	64 366 (10.7)	−1.3 (−1.3 to −1.2)
60-69	7 946 320 (8.3)	35 734 (17.4)	9.1 (9.0 to 9.3)	104 909 (17.4)	9.1 (9.0 to 9.2)
70-79	5 378 153 (5.6)	39 283 (19.1)	13.5 (13.4 to 13.7)	130 483 (21.6)	16.0 (15.9 to 16.1)
≥80	5 318 150 (5.5)	73 575 (35.8)	30.3 (30.1 to 30.5)	242 431 (40.2)	34.7 (34.5 to 34.8)
Invalid or missing	56 802 (0.1)	108 (0.1)	0	147 (<1)	0
Sex					
Female	53 906 302 (56.0)	90 614 (44.1)	−11.9 (−12.1 to −11.7)	290 505 (48.2)	−7.9 (−8.0 to −7.7)
Male	42 259 752 (43.9)	114 582 (55.8)	11.9 (11.7 to 12.1)	312 351 (51.8)	7.9 (7.7 to 8.0)
Unknown	73 885 (0.1)	176 (0.1)	0	417 (0.1)	0
Race					
African American	20 933 184 (21.8)	29 265 (14.2)	−7.5 (−7.7 to −7.3)	63 983 (10.6)	−11.1 (−11.2 to −11.1)
Asian	1 173 569 (1.2)	2189 (1.1)	−0.2 (−0.2 to −0.1)	6830 (1.1)	−0.1 (−0.1 to 0.1)
White	63 834 065 (66.3)	152 672 (74.3)	8.0 (7.8 to 8.2)	486 032 (80.6)	14.2 (14.1 to 14.3)
Other or unknown^a^	10 299 121 (10.7)	21 246 (10.3)	−0.4 (−0.5 to −0.2)	46 438 (7.1)	−3.0 (−3.1 to −2.9)
Ethnicity					
Hispanic	8 793 518 (9.1)	5923 (2.9)	−6.3 (−6.3 to −6.2)	22 889 (3.8)	−5.3 (−5.4 to −5.3)
Not Hispanic	80 613 396 (83.8)	167 387 (81.5)	−2.3 (−2.4 to −2.1)	513 788 (85.2)	1.4 (1.3 to 1.5)
Unknown	6 833 025 (7.1)	32 062 (15.6)	8.5 (8.4 to 8.7)	66 596 (11.0)	3.9 (3.9 to 4.0)
US Census region					
Midwest	47 619 170 (49.5)	99 324 (48.4)	−1.1 (−1.3 to −0.9)	276 130 (44.8)	−3.7 (−3.8 to −3.6)
Northeast	10 964 699 (11.4)	34 473 (16.8)	5.4 (5.2 to 5.6)	77 971 (12.9)	1.5 (1.4 to 1.6)
South	24 362 335 (25.3)	52 997 (25.8)	0.5 (0.3 to 0.7)	23 403 (3.9)	−21.4 (−21.5 to −21.4)
West	7 793 720 (8.1)	10 524 (5.1)	−3.0 (−3.1 to −2.9)	156 273 (25.9)	17.8 (17.7 to 17.9)
Other or unknown	5 500 015 (5.7)	8054 (3.9)	−1.8 (−1.9 to −1.7)	69 496 (11.5)	5.8 (5.7 to 5.9)

Abbreviation: ED, emergency department.

^a^ Other includes American Indian or Alaska Native; Native Hawaiian or Other Pacific Islander.

**Table 3.** Comorbidities of ED Decedents Compared With a Sample of ED Patients Overall, as Categorized by Charlson Comorbidity Index

Characteristic	Participants, No. (%)				
	ED encounter sample (n = 4 811 997)	ED decedents (n = 205 372)	Difference in Proportions vs ED patients overall, mean % (95% CI)	Optum patients with death ≤1 mo of ED visit (n = 603 273)	Difference in Proportions vs ED patients overall, mean % (95% CI)
Congestive heart failure	271 071 (5.6)	51 631 (25.1)	19.5 (19.3-19.7)	188 725 (31.3)	25.7 (25.5-25.8)
Myocardial infarction	189 219 (3.9)	28 738 (14.0)	10.1 (9.9-10.2)	101 679 (16.9)	12.9 (12.8-13.0)
Peripheral vascular disease	252 587 (5.2)	36 826 (17.9)	12.7 (12.5-12.8)	148 605 (24.6)	19.4 (19.3-19.5)
Stroke	257 816 (5.4)	34 699 (16.9)	11.5 (11.4-11.7)	136 975 (22.7)	17.3 (17.2-17.5)
Dementia	69 872 (1.5)	21 211 (10.3)	8.9 (8.7-9.0)	78 379 (13.0)	11.5 (11.5-11.6)
Chronic obstructive pulmonary disease	1 017 594 (21.1)	59 819 (29.1)	8.0 (7.8-8.2)	225 555 (37.4)	16.2 (16.1-16.4)
Connective tissue disease	96 724 (2.0)	7136 (3.5)	1.5 (1.4-1.5)	30 152 (5.0)	3.0 (2.9-3.0)
Peptic ulcer disease	89 119 (1.9)	7308 (3.6)	1.7 (1.6-1.8)	32 050 (5.3)	3.5 (1.8-3.5)
Liver disease					
Mild	248 933 (5.2)	17 527 (8.5)	3.4 (3.2-3.5)	82 510 (13.7)	8.5 (8.4-8.6)
Severe	29 429 (0.6)	4512 (2.2)	1.6 (1.5-1.6)	23 263 (3.9)	3.2 (3.2-3.3)
Diabetes					
Uncomplicated	548 727 (11.4)	50 997 (24.8)	13.4 (13.2-13.6)	180 529 (29.9)	18.5 (18.4-18.6)
With complications	195 865 (4.1)	22 683 (11.0)	7.0 (6.8-7.1)	89 385 (14.8)	10.7 (10.7-10.8)
Chronic kidney disease	258 546 (5.4)	43 855 (21.4)	16.0 (15.8-16.2)	172 580 (28.6)	23.2 (23.1-23.4)
Cancer					
Local	209 690 (4.4)	34 809 (16.9)	12.6 (12.4-12.8)	160 565 (26.6)	22.3 (22.1-22.4)
Metastatic	51 908 (1.1)	15 528 (7.6)	6.5 (6.4-6.6)	82 326 (13.6)	12.6 (12.5-12.7)
AIDS	16 386 (0.3)	666 (0.3)	0	2547 (0.4)	0.1 (0.1-0.1)
Hemiparesis	59 246 (1.2)	6613 (3.2)	2.0 (1.9-2.1)	26 837 (4.4)	3.2 (3.2-3.3)
Total Charlson Comorbidity Index score, median (IQR)	1 (0-3)	6 (4-8)	5 (4-6)	7 (5-9)	6 (5-7)

Abbreviation: ED, emergency department.

**Figure.**
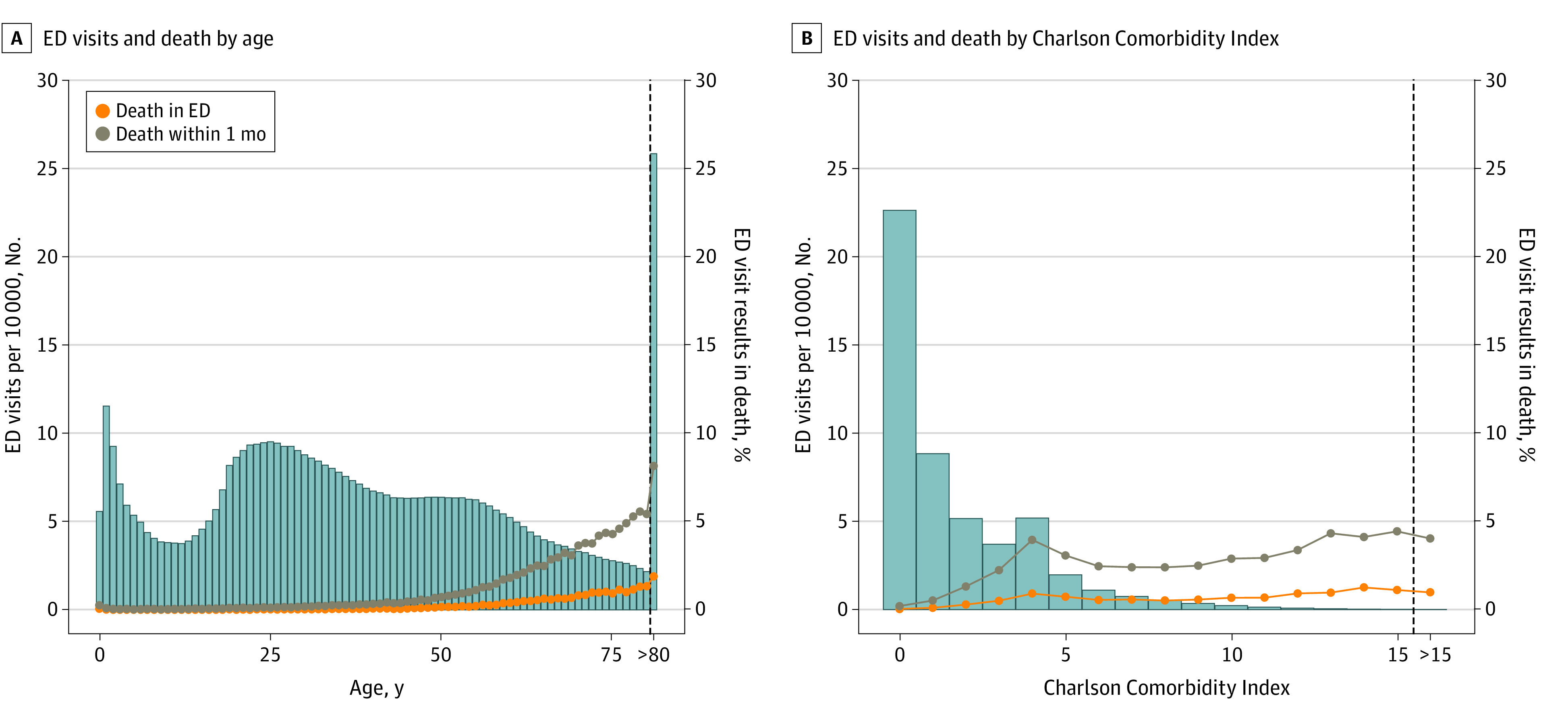
Frequency of Emergency Department (ED) Encounters, Deaths in the ED, and Deaths Within 1 Month of ED Encounter, Stratified by Patient Age and Charlson Comorbidity Index

Estimates of national ED deaths are in Table 1. ED deaths accounted for an estimated 3 018 916 (11.3%) of total deaths from 2010 to 2019 (Table 1). The proportion of total deaths occurring in the ED decreased by 0.27% annually (*P* for trend = .003). In comparison to the total US population, there were 1.01 ED deaths per 1000 residents in 2011. The percentage of ED encounters resulting in death decreased by 0.11% annually (*P* for trend = .004) among patients with congestive heart failure and fell on average 0.04% annually (*P* for trend = .006) among patients with chronic obstructive pulmonary disease. Overall, 29 385 deaths (14.3%) occurred among ED boarders who died after admission to the hospital but before physical departure from the ED. According to our estimates and US Census data, from 2010 to 2019, 33.2% of all decedents visited the ED within 1 calendar month of their death. This proportion increased by 1.2% annually (*P* for trend < .001).

## Discussion

In this cross-sectional study, we found that more than 1 in 3 US residents come to the ED within 1 month of their death, a proportion that has increased 42% over a decade. Moreover, nearly 300 000 US residents die in the ED annually. Taken together, these findings highlight a pressing need to develop systems and resources to support end-of-life care in the ED. Unsurprisingly, we find that patients with death proximate to their final ED encounter are older and have significantly more comorbidities compared with the overall ED population. Among ED encounters for patients older than 80 years of age, nearly 1 in 12 will die within 1 month of that encounter. These patients may exhibit identifiable trajectories of dying that offer an opportunity to avoid unwanted aggressive care or hospitalization at end of life.^10,11,12^ In current practice, these patients often receive life-extending treatments and are admitted to the hospital.^13^

Our findings are generally consistent with past research from other national data sources. Using National Hospital Ambulatory Medical Care Survey (NHAMCS) data from 1997 to 2011, Kanzaria et al^1^ identified a decline in the incidence of death in the ED over time. In 2011, NHAMCS estimated 0.77 ED deaths per 1000 US residents.^1^ Using Optum data, we estimate 1.01 ED deaths per 1000 that same year. This difference may be due to different hospital sampling strategies or operational definitions. For example, because Optum allows identification of a patient’s physical location in the hospital, we chose to include patients who died in the ED after admission who would not be included in research from other administrative data sources. Analyzing a cohort of older US residents and Medicare claims data from 1992 to 2006, Smith et al^3^ found half of decedents had an ED encounter in the last month of life, which is consistent with our findings that one-third of persons overall and that more older persons visit the ED within 1 month of death, In fact, nearly 1 in 12 ED encounters for patients older than 80 years precedes death within 1 month.

There is general consensus that delivering high-quality end-of-life care in the ED is an unmet need.^14^ Compared with dying at home or in a hospice facility, quality of dying in the hospital is worse and may lead to protracted grief and psychological distress for families.^4^ Time constraints, patient volume, and environmental factors may contribute to suboptimal patient care and family experience.^15^ Development and implementation of policies, structural changes, and allocation of additional resources can improve delivery of care to these patients.^2^ ED programs should clearly include grief and bereavement resources. Practitioners and staff in the ED should have core primary palliative care skills, including symptom management for actively dying patients, the ability to give serious news, and focus on talking to patients and families about goals of care. The ED also is the admission pathway for most hospitalized patients. Therefore, optimizing end-of-life care could not only improve the ED environment itself but also avert hospital admission and in-hospital death by building outpatient or home options. ED practitioners and systems of care should consider developing relations with community palliative care and hospice programs to follow up with the large number of patients who, although they will survive their acute encounter, have impending or ongoing palliative care needs. We found that advanced age was the greatest demographic factor associated with risk for ED mortality. This observation can be used to help target resources to support end-of-life care to patients and departments most likely to benefit, while minimizing disruption to the overall mission of the ED to preserve and restore health for the vast majority of patients.

### Limitations

Our work has important limitations. The Optum EHR data set includes detailed patient-level information regardless of patient age and insurance status, and includes data elements not available in other claims-based or other administrative data such as postacute (ie, outpatient) vital status. The demographics and comorbidities of patients in our study resembled the ED population from other broader data sets.^16^ However, hospital systems that contribute data to Optum may not be nationally representative. Thus, our estimates may be biased if, within year and US Census region, Optum-participating hospitals are not representative of overall hospitals within that stratum. To maintain full deidentification, Optum EHR provides the month and year of patient death, but not the exact day. To this end, it also does not include an ED disposition category corresponding to death. We used indirect approaches to identify ED decedents. Although we took precautions to minimize classification error, we may have incorrectly coded some patients as having died in the ED when instead they died later that calendar month.

## Conclusions

In summary, death during or shortly after ED encounters is common, especially among patients who are older and with chronic comorbidities. Although death may occur unexpectedly, many patients will exhibit trajectories that portend imminent death. Robust systems of emergency care must not only offer life-prolonging interventions, but also identify patients and families for whom end-of-life care is necessary or preferred. The frequency with which these scenarios arise suggests a significant public health impact from policies and resources to support delivery of this care in the ED.
